# The I. H. A.

**Published:** 1883-02

**Authors:** 


### HOMŒOPATHIC PHYSICIAN,

A MONTHLY JOURNAL OF MEDICAL SCIENCE.

"If our school ever gives up the strict inductive method of Hahnemann, we are lost, and deserve only to be mentioned as a caricature, in the history of medicine."—CONSTANTINE HERING.

Vol. III. FEBRUARY, 1883. No. 2.

### EDITORIAL.

THE I. H. A.—Our esteemed contemporary, the *Counselor*, has been much disturbed recently over the short-comings of the I. H. A. That Association has, in the *Counselor's* opinion, "talked much and done little." To a certain degree this opinion is correct; for, while some very fine papers have been presented to the Association at its two sessions, there has not been shown by the members in general that disposition to get down to real work which we had hoped to see; to map out a course of work which, earnestly pursued, would interest and benefit the members. Only such work can keep the Association alive or give satisfactory reason for its existence.

The truth of the case may be briefly stated: In its earlier years the American Institute did splendid work for Homœopathy—work which improved and interested its members. And had the Institute continued in such work, no excuse could have been found for a separate organization. Of late years the Institute has not only ceased its good work, but is doing much to destroy the great work done by its founders. Therefore, those who still believed in the Homœopathy of Hahnemann were compelled either to form a separate society or to have their usefulness destroyed by the overwhelming majority of eclectic members of the Institute. This, then, was the reason for the formation of the I. H. A.—to continue the work begun, and carried on so well, by the founders of the American Institute, a work which the Institute has in its later years so sadly neglected. Is this schism?

The founders of the American Institute thus defined the objects of *their* organization:

WHEREAS, A majority of the allopathic physicians continue to deride and oppose the contributions to the materia medica that have been made by the homœopathic school; and whereas, the state of the materia medica in both schools is such as imperatively to demand a more satisfactory arrangement and greater purity of observation, which can only be obtained by associate action on the part of those who diligently *seek for truth alone*,* and, inasmuch as the state of public information respecting the principles and practice of Homœopathy is so defective as to make it easy for *mere pretenders* to this very difficult branch of the healing art to acquire credit as proficients in the same; therefore

*Resolved*, That it is deemed expedient to establish a society entitled "The American Institute of Homœopathy," and the following are declared to be the essential purposes of said Institute:

First. The reformation and augmentation of the materia medica.

Second. The restraining of physicians from pretending to be competent to practice Homœopathy who have not studied it in a careful and skillful manner.

Such was the organization and such were the original purposes of the American Institute. First, to enlarge and purify the materia medica, and secondly, to see that none practiced Homœopathy who had not "studied it in a careful and skillful manner." Two noble purposes! How have they been carried out? Has the American Institute always striven to accomplish these "essential purposes" of its organization? Are its sessions and work devoted to the "reformation and augmentation of the materia medica"? Are the majority of its members those who have "studied Homœopathy in a careful and skillful manner"? These queries must be answered in the negative. Is it, then, a *great sin* for such physicians as so desire to organize in order to pursue this neglected work? Is it schism?

The I. H. A., having been formed to carry on this work, should speedily set about doing it, and not "talk" so much. There has been an abundance of "resolutions," enough for several associations. But, beyond a few individuals, there has not been shown the proper disposition to work. Each individual member should make it his (or her) duty to work for the Association. It is his duty, and cannot be shifted to other shoulders. It is only by earnest, persistent work that the fate depicted by the *Counselor* can be avoided. There may be minor points on which members of the I. H. A. differ, but

* Italics ours.—EDITOR.

they *all* agree upon the essential principles of Homœopathy, and for these they can all *work*.

The duty incumbent upon this Association has been clearly placed before its members by their first president, whose earnest words we quote:

"What, then, are the members of this Association to do, the results of which will justify their existence as an associated body? We know of but one thing, and that is, *work*—earnest, honest, incessant work. *Not work upon partialists, mixed, or old-school men*, but on the elements of sickness, that a knowledge of them in their totality, as we have shown to be necessary in order that an intelligent treatment of them practically may the more readily be obtained when needed; and upon the materia medica, that its elements may be mastered in the same detailed totality, in order that when the simillimum for a cure is needed it may be more readily found, and applied with that certainty of assurance of which guessing makes no part. Work of this sort, persisted in, will by and by mature a power greater than any argument, however masterly, or than any controversy, no matter with what earnestness it may be waged. Work of this sort will in time, by its results, so demonstrate to the public mind the superiority of the pure practice of the Homœopathy we advocate over that which is partial or mixed, as well as over that of the old-school, that these gentlemen, recognizing the education the public has thus received, and at the same time the confidence of the public they themselves have lost, are not in the least danger of neglecting to make haste to claim their share of the honor a numbering with those thus diligent is sure to confer. Thus, and thus only, can the interests of true Homœopathy be advanced, and the objects for which this Association was organized be secured. And in the results of such work only will true Homœopathy find its just illustration which you, gentlemen, by your Association, stand pledged to give to the world."

Let this sound counsel be promptly acted on, and the I. H. A. will be an honor to Homœopathy. Let it be persistently neglected, and the I. H. A. will exist only as an object of ridicule.

